# Exposure to Secondhand Smoke and Secondhand E-Cigarette Aerosol Among Middle and High School Students

**DOI:** 10.5888/pcd16.180531

**Published:** 2019-04-04

**Authors:** Andrea S. Gentzke, Teresa W. Wang, Kristy L. Marynak, Katrina F. Trivers, Brian A. King

**Affiliations:** 1 Office on Smoking and Health, National Center for Chronic Disease Prevention and Health Promotion, Centers for Disease Control and Prevention, Atlanta, Georgia

## Abstract

**Introduction:**

Youth exposure to secondhand smoke (SHS) and secondhand aerosol from e-cigarettes (SHA) may contribute to the renormalization of tobacco product use behaviors. Our study assessed self-reported SHS or SHA exposures in indoor or outdoor public places among US students.

**Methods:**

Data came from the 2015 and 2017 National Youth Tobacco Survey, a school-based survey of US students in grades 6 through 12. Past 30-day exposure to SHS and SHA in indoor and outdoor public places was assessed. The prevalence of exposure was assessed overall and by covariates for each year. We used adjusted prevalence ratios (aPRs) to assess determinants of exposure.

**Results:**

We observed no significant change from 2015 through 2017 in exposure to SHS (52.6% to 50.5%), SHA (25.2% to 25.6%), or either SHS or SHA (56.7% to 55.1%). Following multivariable adjustment, in 2017, exposure to either SHS or SHA in public was higher among female students versus male students (aPR = 1.29), high school students versus middle school students (aPR = 1.15), current e-cigarette users versus nonusers (aPR = 2.89), and current users of other tobacco product versus nonusers (aPR = 1.21). Exposure was higher for students who reported that a household member used tobacco products.

**Conclusion:**

In 2017, more than half (55.1%, 14.3 million) of US middle and high school students reported exposure to secondhand tobacco product emissions in indoor or outdoor public places. E-cigarette use may complicate the enforcement of existing smoke-free policies and contribute to the renormalization of tobacco use behaviors. Continued efforts are warranted to reduce the social acceptability of tobacco product use and protect bystanders from all tobacco product emissions.

**Summary**

**What is already known about this topic?**

Most comprehensive smoke-free policies were enacted before the rise in e-cigarette popularity and do not specifically include e-cigarettes.

**What is added by this report?**

From 2015 to 2017, no change in exposure to secondhand emissions from combustible or electronic tobacco products in indoor or outdoor public places was observed among US youth. In 2017, over 14.3 million youth were exposed to secondhand emissions from tobacco products in public places.

**What are the implications for public health practice?**

Fully enforced, comprehensive, smoke-free policies for indoor environments that include both combustible and electronic tobacco products can reduce the social acceptability of tobacco product use and protect bystanders from all tobacco product emissions.

## Introduction

The adverse health effects of secondhand smoke (SHS) exposure from combustible tobacco products are well established (1). SHS exposure causes sudden infant death syndrome, respiratory infections, ear infections, and more frequent and severe asthma attacks among children, and coronary heart disease, stroke, and lung cancer in adult nonsmokers (1,2). There is no risk-free level of SHS exposure (2). Despite progress in reducing SHS exposure in the United States, an estimated 58 million Americans remain exposed, nearly half of whom (25 million) are aged 3 to 19 (3).

Eliminating smoking in indoor spaces fully protects nonsmokers from SHS exposure in these environments. As of August 2018, 27 states and more than 900 municipalities had implemented comprehensive smoke-free laws that prohibit smoking in indoor public places, including workplaces, restaurants, and bars (4,5). These policies protect bystanders from exposure to SHS and may influence social norms by reducing the social acceptability of tobacco use (6).

Recently, the tobacco product landscape has evolved to include novel products, including e-cigarettes (7). In contrast to smoke from combustible tobacco products, e-cigarette aerosol is only produced during activation of the device (7). Secondhand aerosol (SHA) generally contains fewer toxicants than SHS (7). However, the US Surgeon General concluded that e-cigarette aerosol is not harmless (7). SHA may expose bystanders to harmful constituents such as nicotine, heavy metals, ultrafine particulates, volatile organic compounds, and other toxicants (8,9).

Most comprehensive smoke-free policies were enacted before the rise in e-cigarette popularity and do not specifically include e-cigarettes (10). E-cigarette use in places that otherwise prohibit combustible tobacco products may threaten social norms toward smoke-free policies (11), resulting in confusion about combustible tobacco use and complications with enforcement and compliance (12). SHS exposure in public places was last estimated in 2013 and SHA in 2015; by those estimates, 40% of youth reported past 7-day exposure to SHS (13), and 25% reported past 30-day exposure to SHA (14). Our study reports changes in the prevalence of self-reported exposure to SHS and SHA in indoor and outdoor public places from 2015 through 2017 among US youth.

## Methods

### Data source

Data on SHA and SHS came from the 2015 and 2017 National Youth Tobacco Survey (NYTS), an annual cross-sectional, paper-and-pencil survey administered to US students enrolled in grades 6 through 12. NYTS uses a 3-stage cluster sampling design to provide a nationally representative sample of students attending public and private schools. The sample sizes and overall response rates (RRs) were 17,711 (RR = 63.4%) in 2015 and 17,872 (RR = 68.1%) in 2017. Details about NYTS methods are available elsewhere (15). We report on secondary analysis of de-identified, publicly available data; therefore, no ethics approval was sought.

### Outcome measures

**Secondhand smoke exposure.** Respondents were asked, “During the past 30 days, on how many days did you breathe the smoke from someone who was smoking tobacco products in an indoor or outdoor public place? Examples of indoor public places are school buildings, stores, restaurants, and sports arenas. Examples of outdoor public places are school grounds, parking lots, stadiums, and parks.” Response options were 0 days, 1 or 2 days, 3 to 5 days, 6 to 9 days, 10 to 19 days, 20 to 29 days and all 30 days. Respondents who recorded a response other than 0 days were considered exposed to SHS.

**Secondhand aerosol exposure.** Respondents were asked, “During the past 30 days, on how many days did you breathe the vapor from someone who was using an e-cigarette in an indoor or outdoor public place? Examples of indoor public places are school buildings, stores, restaurants, and sports arenas. Examples of outdoor public places are school grounds, parking lots, stadiums, and parks.” Response options were 0 days, 1 or 2 days, 3 to 5 days, 6 to 9 days, 10 to 19 days, 20 to 29 days and all 30 days. Respondents who recorded a response other than 0 days were considered exposed to SHS.

**Patterns of exposure to secondhand smoke and secondhand aerosol.** To describe patterns of recent (past 30 days) exposure to SHS and SHA in indoor or outdoor public places, 4 additional outcomes were calculated. Students who reported exposure to SHS or SHA, either alone or in combination, were categorized as being exposed to either SHS or SHA. Those who reported SHS exposure, but no SHA exposure, were categorized as reporting exposure to SHS only. Those who reported SHA exposure, but no SHS exposure, were categorized as reporting exposure to SHA only. Finally, students who reported SHS exposure and SHA exposure were categorized as exposed to both SHS and SHA. Respondents indicating no recent exposure to SHS or SHA were the reference group for these 4 outcomes.

### Covariates 

Assessed covariates were sex (male, female), school level (middle school [grades 6–8], high school [grades 9–12]), race/ethnicity (non-Hispanic white, non-Hispanic black, Hispanic, non-Hispanic other), current (past 30 days) use of e-cigarettes (no, yes), current (past 30 days) use of other tobacco products (cigarettes, cigars, smokeless tobacco [chewing tobacco, snus, dissolvable tobacco products], pipe tobacco, hookah, or bidis) (no, yes), and tobacco product use by a household member (no tobacco product use, combustible tobacco product use only [cigarettes, cigars, pipe, hookah, or bidis], smokeless tobacco product use only [chewing tobacco, snus, or dissolvable tobacco products], e-cigarette use only, or use of a combination of combustible, smokeless, and electronic tobacco products).

### Analysis

Data were analyzed using R version 3.2.3 (The R Foundation) and R Survey Package version 3.30–3 to account for the complex sampling design (16). All analyses were conducted on weighted data to provide nationally representative estimates. In 2015 and 2017, the prevalence and corresponding 95% confidence interval of each outcome was calculated overall and stratified by covariates. The 2015 NYTS was the first survey wave to assess youths’ exposure to SHS and SHA in indoor or outdoor public places. Significant differences in each outcome between 2015 and 2017 were assessed by using generalized linear models (*P* < .05). Population counts of exposure were estimated from extrapolated probability weights and rounded down to the nearest 10,000 persons. In 2017, adjusted prevalence ratios (aPRs) of exposure were calculated by using binary logistic regression models with predictive margins to assess the association between each outcome (relative to no exposure) and each covariate.

## Results

### Any SHS and SHA exposure

No significant differences in the overall prevalence of indoor or outdoor exposure to either SHS or SHA, any SHS, or any SHA were observed between 2015 and 2017 (Table 1). In 2015, 56.7% of students (14.7 million) reported exposure to either SHS or SHA, 52.6% (13.7 million) reported exposure to SHS, and 25.2% (6.6 million) reported exposure to SHA in a public place on one or more of the past 30 days. In 2017, 55.1% of students (14.3 million) reported exposure to either SHS or SHA, 50.5% (13.2 million) reported exposure to SHS, and 25.6% (6.7 million) reported exposure to SHA in a public place on one or more of the past 30 days.

**Table 1:** Prevalence of Self-Reported Exposure to Secondhand Tobacco Smoke or Secondhand Aerosol From E-Cigarettes in Public Places During the Past 30 Days Among US Middle and High School Students, National Youth Tobacco Survey, 2015 and 2017^a^

Characteristic	SHS Exposure^b^		SHA Exposure^c^		Exposure to Either SHS or SHA^d^	
	2015	2017	2015	2017	2015	2017
**Overall**	52.6 (50.6–54.5)	50.5 (48.6–52.4)	25.2 (23.5–27.0)	25.6 (23.7–27.0)	56.7 (54.7–58.6)	55.1 (53.2–57.0)
**Sex**						
Male	45.1 (43.0–47.1)	43.2 (41.0–45.4)	23.1 (21.2–25.0)	22.6 (20.5–24.8)	50.0 (47.8–52.1)	48.4 (46.1–50.7)
Female	60.4 (58.2–62.5)	58.1 (55.7–60.4)	27.5 (25.5–29.5)	28.5 (26.4–30.7)	63.7 (61.4–65.9)	62.1 (59.8–64.4)
**School level**						
Middle school (grades 6–8)	47.2 (44.7–49.6)	45.2 (42.7–47.8)	18.8 (17.4–20.4)	20.0 (18.2–21.8)	50.6 (48.0–53.1)	48.8 (46.3–51.3)
High school (grades 9–12)	56.8 (54.4–59.1)	54.6 (52.3–56.8)	30.2 (27.8–32.7)	29.9 (27.3–32.5)	61.5 (59.1–63.8)	60.0 (57.6–62.4)
**Race/ethnicity**						
Non-Hispanic white	57.6 (55.1–60.0)	54.9 (53.0–56.8)	27.7 (25.4–30.1)	29.1 (26.7–31.6)	61.7 (59.0–64.3)	60.2 (58.4–61.9)
Non-Hispanic black	43.1 (40.0–46.2)	38.8 (35.5–42.2)	16.3 (13.8–19.0)	14.5 (12.8–16.3)	46.7 (43.3–50.1)	41.8 (38.5–45.2)
Hispanic	47.1 (44.3–50.1)	48.2 (45.0–51.3)	25.9 (24.0–28.0)	25.5 (23.2–27.9)	51.9 (48.9–54.9)	52.8 (49.4–56.1)
Non-Hispanic other	53.5 (48.9–58.1)	49.5 (44.5–54.5)	22.6 (17.7–28.0)	20.3 (16.5–24.4)	56.7 (52.3–61.6)	52.5 (47.2–57.7)
**E-cigarette use^e^**						
Not current user	50.2 (48.2–52.2)	48.6 (46.5–50.8)	19.4 (18.0–20.8)	21.1 (19.5–22.7)	53.3 (51.2–55.3)	52.4 (50.3–54.5)
Current user	71.4 (68.2–74.5)	71.2 (67.5–74.8)	70.9 (68.2–73.6)	76.5 (73.5–79.3)^f^	83.9 (81.5–86.2)	85.8 (83.5–87.9)
**Other tobacco product use^g^**						
Not current user	49.7 (47.6–51.8)	48.2 (46.1–50.3)	20.9 (19.3–22.6)	22.5 (20.6–24.5)	53.4 (51.2–55.6)	52.6 (50.5–54.7)
Current user	72.0 (69.7–74.2)	72.0 (69.6–74.4)	54.7 (50.6–58.8)	54.9 (51.2–58.5)	78.9 (76.6–81.1)	79.5 (77.6–81.2)
**Tobacco product use by household member(s)** h						
No tobacco products	43.0 (40.6–45.4)	43.1 (40.7–45.6)	18.9 (17.3–20.5)	21.0 (19.0–23.3)	46.5 (44.0–49.0)	46.9 (44.5–49.4)
Combustible only	67.6 (65.7–69.4)	63.6 (61.1–66.1)^f^	24.6 (22.4–26.9)	23.4 (21.1–25.7)	70.7 (68.8–72.5)	66.7 (64.2–69.2)^f^
Smokeless only	50.2 (44.7–55.7)	53.6 (48.1–59.0)	31.1 (26.7–35.7)	30.1 (25.6–35.0)	55.5 (50.5–60.3)	59.0 (53.9–64.0)
E-cigarettes only	50.8 (44.4–57.1)	52.0 (46.5–57.4)	67.2 (60.8–73.2)	77.6 (73.3–81.6)^d^	76.0 (70.0–81.4)	81.8 (77.2–85.9)
Other combination	83.3 (80.9–85.6)	77.2 (74.3–79.9)^f^	57.9 (53.4–62.3)	52.7 (46.9–58.4)	88.0 (85.8–90.0)	84.2 (81.4–86.7)^f^

Abbreviations: SHA, secondhand aerosol; SHS, secondhand smoke.

a 2015: n = 17,711; 2017: n = 17,872. Values are percentage (95% confidence interval).

b A response other than 0 days to the question, “During the past 30 days, on how many days did you breathe the smoke from someone who was smoking tobacco products in an indoor or outdoor public place?” Respondents with missing data (2015, n = 809; 2017, n = 819) were excluded.

c A response other than 0 days to the question, “During the past 30 days, on how many days did you breathe the vapor from someone who was using an electronic cigarette or e-cigarette in an indoor or outdoor public place?” Respondents with missing data (2015, n = 791; 2017, n = 811) were excluded.

d Respondents who reported exposure to either secondhand tobacco smoke or secondhand e-cigarette aerosol during the past 30 days. Respondents with missing data (2015, n = 924; 2017, n = 896) were excluded.

e Current users reported use on ≥1 days of the past 30 days; noncurrent users reported use on 0 days.

f Represents a significant difference in estimates between 2015 and 2017 based on generalized linear modeling (*P* < .05).

g Other tobacco products were cigarettes; cigars, cigarillos, or little cigars; chewing tobacco, snuff, or dip; pipe filled with tobacco; bidis; snus; dissolvable tobacco; or hookah or waterpipe. Current users reported use of ≥1 of these products on ≥1 days of the past 30-days. Noncurrent users reported using all tobacco products on 0 days.

h No tobacco products = no use of tobacco products by a household member; combustible tobacco products only = reported use by a household member of cigarettes, cigars, little cigars, cigarillos, pipe, hookah, or bidis; smokeless tobacco products only = reported use by a household member of chewing tobacco, snuff or dip, snus, or dissolvable tobacco products; e-cigarettes only = use by a household member of e-cigarettes; other combination = reported use by a household member of any other combination of combustible, smokeless, or electronic tobacco products. Respondents with missing data (2015, n = 1,000; 2017, n = 1,058) on household member tobacco product use were excluded.

Among students who reported SHS exposure during the past 30 days in 2017, most (47.3%, 6.2 million) reported exposures on 1 to 2 days of the past 30 days, followed by 3–9 days (30.0%, 3.9 million), 10–29 days (12.9%, 1.7 million), and all 30 days (9.8%, 1.3 million). Among those reporting SHA exposure during the past 30 days, 47.6% (3.2 million) reported exposures on 1–2 days, 30.0% (2.0 million) on 3–9 days, 13.3% (0.9 million) on 10–29 days, and 9.1% (0.6 million) on all 30 days (Figure).

**Figure:**
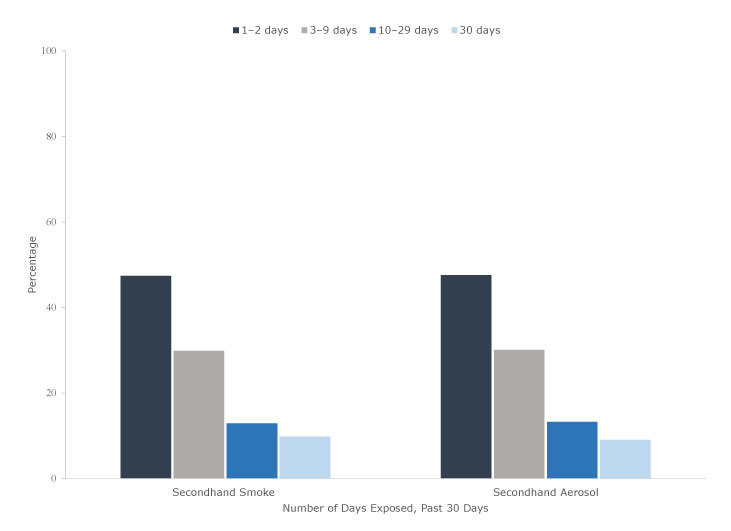
Frequency of self-reported exposure to secondhand smoke and secondhand aerosol from e-cigarettes in public during the past 30 days among US middle and high school students reporting any exposure, National Youth Tobacco Survey, 2017. Left: The frequency of secondhand smoke (SHS) exposure is calculated among respondents who reported being exposed to SHS (unweighted, n = 8,273). Right: The frequency of secondhand aerosol (SHA) exposure is calculated among respondents who reported being exposed to SHA (unweighted, n = 4,138). Percentages are based on weighted data.

**Table:** 

Past 30-Day Exposure (number of days)	Secondhand Smoke (SHS)	Secondhand Aerosol (SHA)	Total Students (SHS) (Weighted N)	Total Students (SHA) (Weighted N)
1–2 days	47.3	47.6	6,220,000	3,170,000
3–9 days	30.0	30.0	3,940,000	2,000,000
10–29 days	12.9	13.3	1,700,000	880,000
30 days	9.8	9.1	1,280,000	600,000

From NYTS 2015 to NYTS 2017, no significant differences in report of any SHS or any SHA exposure were observed by tobacco product use status. Among nonusers of e-cigarettes in 2017, 48.6% (11.6 million) reported SHS exposure and 21.1% (5.0 million) reported SHA exposure during the past 30 days. Among nonusers of other tobacco products in 2017, 48.2% (11.3 million) reported SHS exposure, and 22.5% (5.3 million) reported SHA exposure. In 2017, 85.8% of e-cigarette users (0.7 million) and 79.5% of other tobacco product users (2.0 million) reported exposure to either SHS or SHA during the past 30 days (Table 1).

In 2017, self-reported exposure to any SHS and any SHA was significantly more likely among female students than among male students (SHS, aPR = 1.35; SHA aPR = 1.33), among high school students than among middle school students (SHS, aPR = 1.13; SHA, aPR = 1.20), among current e-cigarette users than among nonusers (SHS, aPR = 1.20; SHA, aPR = 2.90), and among current users of other tobacco products than among nonusers (SHS, aPR = 1.25; SHA, aPR = 1.37). Reports of exposure to any SHS (aPR = 0.75) and any SHA (aPR = 0.65) were lower among non-Hispanic black students than among non-Hispanic white students. Compared with students reporting no tobacco product use by a household member, students reporting family use of only combustible tobacco products increased reporting of SHS exposure in public places (aPR = 1.42). Household member use of only e-cigarettes increased the reports of SHA exposure (aPR = 3.08), and use of a combination of combustible, smokeless, and electronic tobacco products increased the reporting of exposures to SHS (aPR = 1.63) and SHA (aPR = 1.82) (Table 2).

**Table 2:** Correlates of Self-Reported Exposure to Secondhand Smoke and Secondhand Aerosol From E-Cigarettes in Public Places During the Past 30 Days Among US Middle and High School Students, National Youth Tobacco Survey, 2017^a^

Characteristic	SHS Exposure^b^	SHA Exposure^c^	Exposure to Either SHS or SHA^d^	Exposure to SHS Only^e^	Exposure to SHA Only^f^	Exposure to Both SHS and SHA^g^
**Sex**						
Male	Reference					
Female	1.35 (1.29–1.41)^h^	1.33 (1.23–1.43)^h^	1.29 (1.24–1.35)^h^	1.40 (1.32–1.48)^h^	1.22 (1.04–1.41)^h^	1.58 (1.44–1.72)^h^
**School level**						
Middle school (grades 6–8)	Reference					
High school (grades 9–12)	1.13 (1.06–1.20)^h^	1.20 (1.08–1.31)^h^	1.15 (1.08–1.21)^h^	1.16 (1.07–1.25)^h^	1.54 (1.23–1.85)^h^	1.27 (1.13–1.41)^h^
**Race/ethnicity**						
Non-Hispanic white	Reference					
Non-Hispanic black	0.75 (0.68–0.81)^h^	0.65 (0.57–0.72)^h^	0.75 (0.69–0.81)^h^	0.74 (0.66–0.83)^h^	0.61 (0.45–0.77)^h^	0.57 (0.50–0.64)^h^
Hispanic	0.91 (0.85–0.97)^h^	0.97 (0.88–1.05)	0.92 (0.86–0.97)^h^	0.87 (0.79–0.95)^h^	0.95 (0.73–1.16)	0.92 (0.84–1.00)
Non-Hispanic other	0.98 (0.89–1.07)	0.88 (0.75–1.01)	0.96 (0.88–1.04)	0.99 (0.87–1.10)	0.76 (0.48–1.04)	0.90 (0.76–1.03)
**E-cigarette use^i^**						
Not current user	Reference					
Current user	1.20 (1.10–1.30)^h^	2.90 (2.67–3.12)^h^	2.89 (2.67–3.12)^h^	0.79 (0.64–0.94)^h^	4.87 (3.76–5.99)^h^	2.25 (2.04–2.46)^h^
**Other tobacco product use^j^**						
Noncurrent user	Reference					
Current user	1.25 (1.17–1.33)^h^	1.37 (1.23–1.52)^h^	1.21 (1.13–1.29)^h^	1.24 (1.11–1.38)^h^	1.09 (0.81–1.39)	1.49 (1.32–1.66)^h^
**Tobacco product use by household member(s)^k^**						
No tobacco products	Reference					
Combustible, only	1.42 (1.35–1.49)^h^	0.98 (0.89–1.07)	1.35 (1.30–1.41)^h^	1.68 (1.58–1.78)^h^	1.09 (0.87–1.32)	1.30 (1.18–1.42)^h^
Smokeless only	1.09 (0.95–1.22)	1.10 (0.93–1.26)	1.09 (0.97–1.20)	1.13 (0.92–1.34)	1.25 (0.83–1.68)	1.11 (0.91–1.32)
E-cigarettes, only	1.08 (0.96–1.21)	3.08 (2.72–3.44)^h^	1.60 (1.47–1.73)^h^	0.56 (0.26–0.85)^h^	7.46 (5.88–9.03)^h^	2.36 (2.00–2.72)^h^
Other combination	1.63 (1.53–1.74)^h^	1.82 (1.59–2.05)^h^	1.63 (1.53–1.73)^h^	1.92 (1.75–2.10)^h^	3.00 (1.86–4.15)^h^	2.32 (2.07–2.58)^h^

Abbreviations: SHA, secondhand aerosol; SHS, secondhand smoke.

a N = 17,872. Values are adjusted prevalence ratio (95% confidence interval).

b A response other than 0 days to the question, “During the past 30 days, on how many days did you breathe the smoke from someone who was smoking tobacco products in an indoor or outdoor public place?” Respondents with missing data (n = 819) were excluded.

c A response other than 0 days to the question, “During the past 30 days, on how many days did you breathe the vapor from someone who was using an electronic cigarette or e-cigarette in an indoor or outdoor public place?” Respondents with missing data (n = 811) were excluded.

d Respondents who reported exposure to SHS or SHA during the past 30 days.

e Respondents who reported exposure to SHS but no exposure to SHA in past 30 days.

f Respondents who reported exposure to SHA but no exposure to SHS in past 30 days.

g Respondents who reported exposure to SHS and exposure to SHA in past 30 days.

h Significant differences in the estimate compared with the reference group, based on multivariable logistic regression modeling (*P* < .05). Each column represents a separate model of reporting the specified outcome adjusted for each variable listed.

i Current users reported use on ≥1 days of the past 30 days; noncurrent users reported use of e-cigarettes on 0 days.

j Includes cigarettes; cigars, cigarillos, or little cigars; chewing tobacco, snuff, or dip; pipe filled with tobacco; bidis; snus; dissolvable tobacco; or hookah or waterpipe. Current users reported use of one or more of these products on ≥1 days of the past 30-days. Noncurrent users reported using all tobacco products on 0 days.

k No tobacco products = no use of tobacco products by a household member reported; combustible tobacco products, only = reported use by a household member of cigarettes, cigars, little cigars, cigarillos, pipe, hookah, or bidis; smokeless tobacco products only = reported use by a household member of chewing tobacco, snuff or dip, snus, or dissolvable tobacco products; e-cigarettes only = reported use by a household member of e-cigarettes; other combination = reported use by a household member of any other combination of combustible, smokeless, or electronic tobacco products. Respondents with missing data (n = 1,058) on tobacco product use by household member(s) were excluded.

### Exclusive and combined SHS and SHA exposure

No significant differences in the overall prevalence of exposure in a public indoor or outdoor place during the past 30 days to SHS only, SHA only, or both SHS and SHA were observed between 2015 and 2017 (Table 3). In 2015, 21.1% (5.5 million) reported exposure to both SHS and SHA in a public place, 31.5% (8.2 million) reported exposure to SHS only, and 4.1% (1.1 million) reported exposure to SHA only. In 2017, 21.0% (5.4 million) reported exposure to both SHS and SHA in a public place, 29.5% (7.6 million) reported exposure to only SHS, and 4.7% (1.2 million) reported exposure to only SHA.

**Table 3:** Prevalence of Exclusive and Combined Self-Reported Exposure to Secondhand Smoke and Secondhand Aerosol From E-Cigarettes in Public Places During the Past 30 Days Among US Middle and High School Students, National Youth Tobacco Survey, 2015 and 2017^a^

Characteristic	SHS Exposure Only^b^		SHA Exposure Only^c^		Exposure to Both SHS and SHA^d^	
	2015	2017	2015	2017	2015	2017
**Overall**	31.5 (30.2–32.7)	29.5 (27.4–31.5)	4.1 (3.6–4.5)	4.7 (4.1–5.3)	21.1 (19.6–22.7)	21.0 (19.4–22.5)
**Sex**						
Male	26.9 (25.3–28.6)	25.8 (23.8–27.7)	4.8 (4.2–5.6)	5.3 (4.4–6.2)	18.2 (16.6–19.9)	17.4 (15.7–19.1)
Female	36.2 (34.6–37.8)	33.5 (31.0–36.1)	3.3 (2.8–3.8)	4.1 (3.6–4.6)^e^	24.2 (22.3–26.2)	24.5 (22.5–26.5)
**School level**						
Middle school (grades 6–8)	31.7 (30.0–33.4)	28.8 (26.6–31.0)^e^	3.4 (3.0–3.8)	3.6 (3.1–4.2)	15.5 (14.1–16.9)	16.4 (14.8–18.1)
High school (grades 9–12)	31.3 (29.6–33.1)	30.1 (27.6–32.7)	4.6 (3.9–5.4)	5.5 (4.6–6.5)	25.6 (23.4–27.7)	24.4 (22.4–26.5)
**Race/ethnicity**						
Non-Hispanic white	34.0 (32.4–35.6)	31.1 (28.2–34.0)	4.1 (3.6–4.7)	5.3 (4.5–6.2)	23.6 (21.5–25.7)	23.8 (21.8–25.9)
Non-Hispanic black	30.5 (27.8–33.2)	27.2 (24.4–30.2)	3.5 (2.3–4.9)	3.0 (2.3–3.8)	12.8 (11.1–14.6)	11.6 (10.1–13.3)
Hispanic	26.0 (24.1–27.9)	27.2 (24.9–29.6)	4.7 (3.9–5.5)	4.6 (3.5–5.9)	21.3 (19.5–23.1)	20.9 (19.3–22.6)
Non-Hispanic other	33.9 (29.2–38.8)	32.3 (29.2–35.4)	3.1 (1.8–4.8)	3.2 (2.1–4.6)	19.8 (15.4–24.7)	17.0 (13.7–20.7)
**E-cigarette use^f^**						
Noncurrent user	33.8 (32.5–35.2)	31.3 (29.2–33.5)^e^	3.0 (2.7–3.4)	3.8 (3.3–4.3)^e^	16.4 (15.1–17.7)	17.3 (15.9–18.7)
Current user	13.1 (11.4–15.0)	9.3 (7.4–11.4)^e^	12.3 (10.5–14.4)	14.5 (11.7–17.7)	58.5 (55.6–61.3)	62.0 (58.2–65.7)
**Other tobacco product use^g^**						
Not current user	46.6 (31.2–33.8)	30.0 (27.9–32.2)^e^	3.7 (3.2–4.1)	4.4 (3.8–5.0)	17.3 (15.8–18.8)	18.2 (16.6–19.8)
Current user	21.1 (21.7–27.1)	24.6 (21.3–28.1)	6.8 (5.4–8.5)	7.5 (5.9–9.5)	47.7 (44.2–51.3)	47.3 (44.1–50.5)
**Tobacco product use by household member(s)^h^**						
No tobacco products	27.6 (26.0–29.3)	25.9 (24.2–27.6)	3.5 (3.0–4.0)	3.9 (3.3–4.6)	15.4 (13.9–16.9)	17.2 (15.3–19.2)
Combustible only	46.1 (44.0–48.3)	43.3 (40.2–46.4)	2.9 (2.2–3.7)	3.2 (2.5–4.1)	21.6 (19.6–23.7)	20.2 (18.4–22.1)
Smokeless only	24.5 (20.0–29.4)	29.0 (23.1–35.4)	5.4 (3.7–7.5)	5.4 (3.5–7.8)	25.6 (20.9–30.8)	24.7 (19.9–29.8)
E-cigarettes only	8.9 (6.3–12.2)	4.2 (2.4–6.8)^e^	25.2 (20.2–30.8)	29.7 (24.6–35.2)	41.9 (35.5–48.4)	47.9 (42.7–53.0)
Other combination	30.0 (26.6–33.6)	31.4 (26.5–36.6)	4.7 (3.6–6.1)	6.9 (5.0–9.3)	53.3 (48.9–57.7)	45.8 (40.9–50.8)^e^

Abbreviations: SHA, secondhand aerosol; SHS, secondhand smoke.

a 2015: n=17,711; 2017: n=17,872. Values are percentage (95% confidence interval).

b Respondents who reported exposure to SHS on 1 or more of the past 30 days, but reported no exposure to SHA during the past 30 days.

c Respondents who reported SHA exposure on 1 or more of the past 30 days, but no SHS exposure during the past 30 days.

d Respondents who reported exposure to both SHS and SHA on 1 or more of the past 30 days.

e Represents a significant difference in estimates between 2015 and 2017 based on generalized linear modeling (*P* < .05).

f Current users reported use of e-cigarettes on ≥1 days of the past 30 days; noncurrent users reported use of e-cigarettes on 0 days.

g Includes cigarettes; cigars, cigarillos, or little cigars; chewing tobacco, snuff, or dip; pipe filled with tobacco; bidis; snus; dissolvable tobacco; or hookah or waterpipe. Current users reported use of ≥1 of these products on ≥1 days of the past 30 days. Noncurrent users reported using all tobacco products on 0 days.

h No tobacco products = no use of tobacco products by a household member; combustible tobacco products only = reported use by a household member of cigarettes, cigars, little cigars, cigarillos, pipe, hookah, or bidis; smokeless tobacco products only = reported use by a household member of chewing tobacco, snuff or dip, snus, or dissolvable tobacco products; e-cigarettes only = reported use by a household member of e-cigarettes; other combination = reported use by a household member of any other combination of combustible, smokeless, or electronic tobacco products. Respondents with missing data (2015, n = 1,000; 2017, n = 1,058) on tobacco product use by household member(s) were excluded.

Female students were more likely than male students to report exposure to both SHS and SHA (aPR = 1.58); SHS only (aPR = 1.40); and SHA only (aPR = 1.22) (Table 2). Compared with middle school students, high school students were more likely to report exposure to both SHS and SHA (aPR = 1.27); SHS only (aPR = 1.16); and SHA only (aPR = 1.54). Non-Hispanic black students were less likely to report exposures to both SHS and SHA (aPR = 0.57); SHS only (aPR = 0.74); and SHA only (aPR = 0.61) than non-Hispanic white students.

Compared with nonusers, current e-cigarette users were more likely to report exposure to both SHS and SHA (aPR = 2.25) and SHA only (aPR = 4.87), but were less likely to report exposure to SHS only (aPR = 0.79). Current users of other tobacco products were more likely to report exposure to both SHS and SHA (aPR = 1.49) and SHS only (aPR = 1.24). Compared with students reporting no tobacco product use by a household member, use of only combustible tobacco products (aPR = 1.68) or a combination of combustible, smokeless, and electronic tobacco products (aPR = 1.92) increased the likelihood of reporting exposure to SHS only; students reporting household member use of only e-cigarettes (aPR = 0.56) were less likely to report SHS only exposures in public places. Reported exposure to only SHA was more likely among students who reported use of e-cigarettes only (aPR = 7.46) or a combination of tobacco product types (aPR = 3.00) by a household member.

## Discussion

From the 2015 NYTS to the 2017 NYTS, no change in exposure to secondhand emissions from combustible or electronic tobacco products in indoor or outdoor public places was observed overall among US middle and high school students. An estimated 14.3 million students (55.1%) reported exposure to tobacco product emissions in 2017, including approximately 1 in 2 youth (13.2 million) who reported any SHS exposure and 1 in 4 youth (6.7 million) who reported any SHA exposure. These findings underscore the importance of smoke-free policies as part of comprehensive tobacco control programs to address SHS and SHA exposures among US youth.

Strongly enforced and comprehensive smoke-free policies that include e-cigarettes have several important benefits such as the potential to reduce the social acceptability of tobacco product use (6), promote smoking cessation (2), and support efforts to decrease smoking initiation among youth (17). Some e-cigarettes resemble various combustible tobacco products, and exhaled aerosol can look like tobacco smoke, particularly to youth (11). In addition to complicating enforcement of existing comprehensive smoke-free policies (12), use of e-cigarettes in indoor or outdoor places where smoking is otherwise prohibited may contribute to the renormalization of behaviors related to use of tobacco products (11), because adolescents are particularly vulnerable to visual cues and social norms (11,18).

As of January 2, 2019, nearly 60% of the US population was protected by a comprehensive state or local smoke-free policy prohibiting smoking in all indoor public places, including worksites, restaurants, and bars (19). However, most of these policies were implemented before the rise in popularity of e-cigarettes in the US marketplace (10,20). As of January 2, 2019, 11 US states, the District of Columbia, Puerto Rico, and more than 600 localities included e-cigarettes in comprehensive smoke-free policies (10,21). Our study’s findings show that millions of US youth continue to report exposure to tobacco product emissions in public environments. In 2017, more than 1 in 5 youth (5.4 million) reported recent exposures to both SHS and SHA in public places, in addition to over 7.6 million who reported exclusive SHS exposure, and over 1.2 million who reported exclusive SHA exposure. The most recent comprehensive statewide smoke-free policy was implemented in 2010, though momentum has continued at the local level (20). Furthermore, approximately three-quarters of the US population live in states where e-cigarette use is not prohibited in all indoor worksites, restaurants, and bars (22). Thus, our findings reinforce the importance of sustained efforts to implement comprehensive smoke-free policies in indoor environments that protect the public from all forms of tobacco product emissions, including both SHS and SHA.

We observed variations in reported SHS and SHA exposure across population groups. Female students and high school students were more likely to report SHS and SHA exposures in indoor or outdoor public places, whereas non-Hispanic black youth were less likely to report such exposures. Literature demonstrates that the prevalence of SHS exposure, measured by serum cotinine, is higher among non-Hispanic black youth overall (3). This discrepancy may be attributable to the use of biomarkers, which reflect SHS exposures occurring in multiple settings, including public places, homes, vehicles, and other locations. Additionally, nicotine metabolism may be slower among non-Hispanic black populations, contributing to higher exposure levels measured from biomarkers (3). However, racial or ethnic variations in smoke-free policy coverage in other settings may contribute to higher SHS exposures among this population than among other subgroups (3).

Compared to nonusers, current users of e-cigarettes were more likely to report SHA exposure in indoor or outdoor public places, whereas current users of other tobacco products were more likely to report SHS exposures. This finding could reflect the fact that youth may socialize with others who share their tobacco product use behaviors (23). The lack of a change in SHS and SHA exposure from the 2015 NYTS to the 2017 NYTS may reflect changes in tobacco product use among youth and adults occurring during this period. Among youth, use of e-cigarettes and combustible tobacco declined and then plateaued from 2015 to 2017 (24). However, e-cigarette use increased considerably among US youth during 2017–2018, while no changes in combustible tobacco product use were observed (24). Among adults, combustible tobacco product use declined during that period, whereas no changes in e-cigarette use was observed during this same period (25). Thus, changes in the patterns of exposure to SHS or SHA may occur in the future as the tobacco product use landscape continues to evolve. Exposures to SHS only and SHA only, were more likely among students reporting that a household member used exclusively combustible tobacco products or exclusively e-cigarettes. Thus, youth living with tobacco product users may experience greater exposures to SHS and SHA in both private and public locations compared with youth living in homes without tobacco product users (26). Animal studies suggest that chronic exposure to nicotine during adolescence, a critical period of brain development, is associated with long-term effects in reward-seeking behaviors, attention and cognition, mood, and other aspects of brain development (7). Accordingly, exposure to nicotine from secondhand tobacco product emissions might be particularly problematic among youth. This reinforces the importance of indoor environments that are free from SHS and SHA, including private settings such as homes, which remain the primary source of SHS exposure among youth (2).

Our study has limitations. First, data were collected from students enrolled in traditional middle and high schools in the United States, so the findings may not be generalizable to all US youth, particularly those who are homeschooled or not enrolled in school. However, the majority of US youth aged 10 to 13 (98.5%) and 14 to 17 (95.5%) are enrolled in a traditional school (27). Second, our outcomes combine reported exposures to SHS and SHA from indoor and outdoor public places; therefore, it was not possible to distinguish between exposures in these 2 environments. Furthermore, outdoor emissions from tobacco products may be transient or dependent on other environmental conditions that could influence recall of exposures (28). Finally, survey responses were self-reported and exposures could not be validated by using biomarkers. Youth may have difficulties recalling SHS and SHA exposures that occurred during the preceding 30-day period. Furthermore, SHA may lack a distinctive odor because of the range of e-cigarette flavors available (29). Thus, youth may have difficulty identifying SHA in the absence of directly observing e-cigarette use, resulting in an underestimate of exposures. However, biomarkers cannot distinguish between sources or methods of exposure to nicotine-containing tobacco products such as SHS or SHA, current tobacco product use or secondhand exposure, or exposures occurring in various locations. This reinforces the value of self-reported data for informing location-specific exposures to SHS and SHA among youth.

From the 2015 NYTS to the 2017 NYTS, no significant change occurred in exposure to tobacco product emissions among US middle and high school students. In 2017, over 14.3 million youth (55.1%) reported exposure to either SHS or SHA in indoor or outdoor public locations. Moreover, an estimated 1.2 million youth (4.7%) reported being exposed exclusively to SHA. Fully enforced, comprehensive, smoke-free policies for indoor environments that include both combustible and electronic tobacco products are critical to reduce the social acceptability of tobacco product use and to protect bystanders from all tobacco product emissions.

## References

[1] US Department of Health and Human Services. The health consequences of smoking: 50 years of progress. A report of the Surgeon General. Atlanta (GA): US Department of Health and Human Services, Centers for Disease Control and Prevention, National Center for Chronic Disease Prevention and Health Promotion, Office on Smoking and Health; 2014.

[2] US Department of Health and Human Services. The health consequences of involuntary exposure to tobacco smoke: A report of the Surgeon General. Atlanta (GA): US Department of Health and Human Services, Centers for Disease Control and Prevention, Coordinating Center for Health Promotion, National Center for Chronic Disease Prevention and Health Promotion, Office on Smoking and Health; 2006.

[3] Homa DM , Neff LJ , King BA , Caraballo RS , Bunnell RE , Babb SD , ; Centers for Disease Control and Prevention (CDC). Vital signs: disparities in nonsmokers’ exposure to secondhand smoke — United States, 1999–2012. MMWR Morb Mortal Wkly Rep 2015;64(4):103–8. 25654612 PMC4584848

[4] Tynan MA , Holmes CB , Promoff G , Hallett C , Hopkins M , Frick B . State and local comprehensive smoke-free laws for worksites, restaurants, and bars — United States, 2015. MMWR Morb Mortal Wkly Rep 2016;65(24):623–6. 10.15585/mmwr.mm6524a4 27337212

[5] Centers for Disease Control and Prevention (CDC). Smoking and tobacco use. State Tobacco Activities Tracking and Evaluation (STATE) System: map of smokefree indoor air — private worksites, restaurants, and bars. https://www.cdc.gov/statesystem/smokefreeindoorair.html. Updated June 30, 2018. Accessed August 30, 2018.

[6] Satterlund TD , Lee JP , Moore RS . Changes in smoking-related norms in bars resulting from California’s Smoke-Free Workplace Act. J Drug Educ 2012;42(3):315–26. 10.2190/DE.42.3.d 23705511 PMC3671496

[7] US Department of Health and Human Services. E-cigarette use among youth and young adults. A report of the Surgeon General. Atlanta (GA): US Department of Health and Human Services, Centers for Disease Control and Prevention, National Center of Chronic Disease Prevention and Health Promotion, Office on Smoking and Health; 2016.

[8] Czogala J , Goniewicz ML , Fidelus B , Zielinska-Danch W , Travers MJ , Sobczak A . Secondhand exposure to vapors from electronic cigarettes. Nicotine Tob Res 2014;16(6):655–62. 10.1093/ntr/ntt203 24336346 PMC4565991

[9] Schripp T , Markewitz D , Uhde E , Salthammer T . Does e-cigarette consumption cause passive vaping? Indoor Air 2013;23(1):25–31. 10.1111/j.1600-0668.2012.00792.x 22672560

[10] American Nonsmokers’ Rights Foundation. States and municipalities with laws regulating use of electronic cigarettes. https://no-smoke.org/wp-content/uploads/pdf/ecigslaws.pdf. Updated January 2, 2019. Accessed February 6, 2019.

[11] World Health Organization. Electronic nicotine delivery systems: report by WHO. 2014. http://apps.who.int/gb/fctc/PDF/cop6/FCTC_COP6_10-en.pdf?ua=1. Accessed May 2, 2018.

[12] Shi Y , Cummins SE , Zhu SH . Use of electronic cigarettes in smoke-free environments. Tob Control 2017;26(e1, e1):e19–22. 10.1136/tobaccocontrol-2016-053118 27609779 PMC5342954

[13] Agaku IT , Singh T , Rolle I , Olalekan AY , King BA . Prevalence and determinants of secondhand smoke exposure among middle and high school students. Pediatrics 2016;137(2):e20151985. 10.1542/peds.2015-1985 26755696

[14] Wang TW , Marynak KL , Agaku IT , King BA . Secondhand exposure to electronic cigarette aerosol among U.S. youths. JAMA Pediatr 2017;171(5):490–2. 10.1001/jamapediatrics.2016.4973 28319226

[15] Centers for Disease Control and Prevention. Smoking and tobacco use: historical NYTS data and documentation; 2018. https://www.cdc.gov/tobacco/data_statistics/surveys/nyts/data/index.html. Accessed August 30, 2018.

[16] Lumley T . Survey: analysis of complex survey samples. R package version 3.30–3 (Survey archive). http://cran.r-project.org/web/packages/survey/index.html. Published August 31, 2014. Accessed May 2, 2018.

[17] Song AV , Dutra LM , Neilands TB , Glantz SA . Association of smoke-free laws with lower percentages of new and current smokers among adolescents and young adults: an 11-year longitudinal study. JAMA Pediatr 2015;169(9):e152285. 10.1001/jamapediatrics.2015.2285 26348866 PMC4577051

[18] US Department of Health and Human Services. Preventing tobacco use among youth and young adults. A report of the Surgeon General. Atlanta (GA): US Department of Health and Human Services, Centers for Disease Control and Prevention, National Center for Chronic Disease Prevention and Health Promotion, Office on Smoking and Health; 2012.

[19] American Nonsmokers’ Rights Foundation. Summary of 100% smokefree state laws and population protected by 100% US smokefree laws. https://no-smoke.org/wp-content/uploads/pdf/SummaryUSPopList.pdf. Updated January 2, 2019. Accessed February 5, 2019.

[20] American Nonsmokers’ Rights Foundation. Chronological table of the US population protected by 100% smokefree state or local laws. https://no-smoke.org/wp-content/uploads/pdf/EffectivePopulationList.pdf. Updated January 2, 2019. Accessed February 6, 2019.

[21] Centers for Disease Control and Prevention. State Tobacco Activities Tracking and Evaluation (STATE) System. Custom report: legislation, e-cigarette-smokefree indoor air. https://nccd.cdc.gov/STATESystem/rdPage.aspx?rdReport=OSH_State.CustomReports&rdAgReset=True&rdShowModes=showResults&rdShowWait=true&rdPaging=Interactive&islMeasure=670CGR. Accessed August 30, 2018.

[22] Marynak K , Kenemer B , King BA , Tynan MA , MacNeil A , Reimels E . State laws regarding indoor public use, retail sales, and prices of electronic cigarettes — US States, Guam, Puerto Rico, and US Virgin Islands, September 30, 2017. MMWR Morb Mortal Wkly Rep 2017;66(49):1341–6. 10.15585/mmwr.mm6649a1 29240728 PMC5730213

[23] Windle M , Haardörfer R , Lloyd SA , Foster B , Berg CJ . Social influences on college student use of tobacco products, alcohol, and marijuana. Subst Use Misuse 2017;52(9):1111–9. 10.1080/10826084.2017.1290116 28524716 PMC5517128

[24] Gentzke AS , Creamer M , Cullen KA , Ambrose BK , Willis G , Jamal A , Vital Signs: Tobacco Product Use Among Middle and High School Students - United States, 2011-2018. MMWR Morb Mortal Wkly Rep 2019;68(6):157–64. 10.15585/mmwr.mm6806e1 30763302 PMC6375658

[25] Wang TW , Asman K , Gentzke AS , Cullen KA , Holder-Hayes E , Reyes-Guzman C , Tobacco product use among adults — United States, 2017. MMWR Morb Mortal Wkly Rep 2018;67(44):1225–32. 10.15585/mmwr.mm6744a2 30408019 PMC6223953

[26] Arechavala T , Continente X , Pérez-Ríos M , Schiaffino A , Fernandez E , Cortés-Francisco N , Second-hand smoke exposure in homes with children: assessment of airborne nicotine in the living room and children’s bedroom. Tob Control 2018;27(4):399–406. 10.1136/tobaccocontrol-2017-053751 28822971

[27] United Stated Census Bureau. School enrollment in the United States: Table 1: enrollment status of the population 3 years old and over, by sex, age, race, Hispanic origin, foreign born, and foreign-born parentage: October 2016. https://www.census.gov/data/tables/2016/demo/school-enrollment/2016-cps.html. Accessed May 2, 2018.

[28] Licht AS , Hyland A , Travers MJ , Chapman S . Secondhand smoke exposure levels in outdoor hospitality venues: a qualitative and quantitative review of the research literature. Tob Control 2013;22(3):172–9. 10.1136/tobaccocontrol-2012-050493 23220937 PMC3803107

[29] National Academies of Sciences, Engineering, and Medicine. Public health consequences of e-cigarettes. Washington (DC): The National Academies Press, 2018. 29894118

